# Psychological mediators between risk perception and psychological wellbeing in emergency healthcare workers

**DOI:** 10.3389/fpsyg.2024.1435189

**Published:** 2024-10-03

**Authors:** Maria del Carmen Badía, Rafael Alarcón, Javier Trillo, Jesús Miranda-Páez

**Affiliations:** ^1^University of Malaga, Málaga, Spain; ^2^Hospital Comarcal Santa Ana, Granada, Spain; ^3^Department of Psychobiology and Behavioral Sciences Methodology, University of Malaga, Málaga, Spain; ^4^Strategic Chair of Security, Emergencies and Disasters. University of Malaga, Málaga, Spain

**Keywords:** risk perception, psychological wellbeing, healthcare professionals, mediation, analysis

## Abstract

Healthcare workers are subjected to numerous work-related stress factors, which have negative consequences on their physical and mental health, making them a vulnerable group. The recent pandemic caused by the new coronavirus created a high demand for attention from healthcare workers, which put their mental health at risk. This study aimed to test a mediation model in which resilience and the satisfaction of psychological needs play a mediating role in risk perception, the attitude toward the management carried out by the administration, emotional intelligence on psychological wellbeing, and depressive symptoms of frontline professionals. The sample consisted of 405 healthcare professionals aged between 22 and 65 years, belonging to critical care units in southern Spain. Linear correlation and mediation analyses were performed. The results showed that psychological wellbeing had positive correlations with resilience and negative correlations with the discrepancy in the satisfaction of psychological needs. Depressive symptoms had negative correlations with resilience and positive correlations with the satisfaction of psychological needs. To explore these results further, a mediation analysis was conducted, and a large set of significant indirect effects was found.

## 1 Introduction

Healthcare workers are subjected to numerous work-related stressors in their daily work life, which have negative consequences on their physical and mental health, making them a vulnerable group (Biggs et al., 2017; Tong et al., 2022). Physical health is understood as a set of activities, postures, and functions that one must have to maintain an optimal physiological state. Mental health is defined as the state in which human beings understand the need to identify factors that allow them to think, feel, and act toward life, which include emotional, psychological, and social wellbeing (Biggs et al., 2017; Tong et al., 2022; Cénat et al., 2022).

Lazarus and Folkman defined stress as a relational process between the individual and his or her environment, in which particular characteristics of the individual and the nature and demands of the environment are considered. They proposed that the experience of stress is the result of a person’s cognitive evaluation of a situation (Biggs et al., 2017; Tong et al., 2022; Cénat et al., 2022).

The risk of becoming infected by numerous biological agents is recognized as one of the most important risks for personnel providing services in the health field, particularly nursing and medical professionals, as they are the ones who have direct and continuous contact with patients and perform daily care tasks involving procedures of all kinds, thereby exposed to all types of pathogenic agents (Cénat et al., 2022; Seckman, 2023). This exposure is much greater if they work in intensive care units, emergency units, etc., where, in addition to pathogens, there is an added stress burden due to the particularity of these services. A study carried out in a university hospital showed that in these units, there is a moderate perception of work-related stressors amongst the professionals analyzed. In addition, the greatest stressors were the lack of control in decision-making on the part of the professionals, the need to continually learn new things, etc. (Carrillo-García et al., 2016). Another study showed critical levels of stress in these units. The different stressful situations that influence this level of stress include the fear of making a mistake while taking care of a patient, not knowing how specialized equipment works, lack of personnel to adequately cover the service, etc. (Lastre-Amell et al., 2018).

The relevance and attention given to the issue of psychosocial risks at work, to which healthcare workers May be exposed, are growing. In addition to the consequences on their mental and physical health, there are other issues on the quality of working life and the effectiveness of these professionals (Lugo-González et al., 2022; Ato and Vallejo, 2011). The importance of maintaining adequate mental health is fundamental for healthcare workers to exercise and provide quality care to patients (Porras-Povedano and Santacruz-Hamer, 2014). Over the years, and especially after the recent pandemic, numerous studies have focused on the mental health of healthcare workers, making it a priority issue, as numerous symptoms such as depression, anxiety, fear, sleep disturbances, and suicidal ideation have been identified. All these symptoms have a direct impact on how professionals treat patients and make decisions (Ato and Vallejo, 2011; Porras-Povedano and Santacruz-Hamer, 2014; Fang-Huerta Mdl et al., 2015; Erquicia et al., 2020; Mingote Adán and Nuñez, 2011). It is crucial for healthcare workers to possess protective factors for their mental health, such as resilience and emotional intelligence. It is also essential to emphasize the importance of emotional care for healthcare workers (Erquicia et al., 2020; dos Santos et al., 2019; Mingote Adán and Nuñez, 2011; Hernández, 2020).

Resilience is defined by the American Psychological Association (APA) as a process of adapting appropriately in the face of adversity, trauma, tragedy, or threat. It is a learnable skill and it is not regarded as a personality trait (Real et al., 2021; Martinez Arriaga et al., 2021; Arrogante, 2014a; Arrogante, 2015; Caro Alonso and Rodriguez, 2018; De Caneva et al., 2020). It is considered a predictor of good mental, physical, and social health. Fletcher and Sarkar pointed out that, although resilience has been conceptualized in many different ways, most definitions are based on two central aspects: adversity and positive adaptation. Thus, for resilience to be demonstrated, both adversity and positive adaptation must be evident. Adversity usually includes negative life circumstances that are known to be statistically associated with adaptive difficulties (De Caneva et al., 2020; Fletcher and Sarkar, 2013; Bueno Ferrán and Barrientos-Trigo, 2021; Dosil Santamarría et al., 2021; Peñafiel-León et al., 2021).

Another protective factor that must be highlighted is emotional intelligence as the ability of healthcare workers to manage their own emotions is of vital importance for providing better patient care and experiencing greater wellbeing; it also helps in achieving optimal professional growth and development by enabling the workers to make appropriate decisions in critical situations (Ordoñez-Rufat et al., 2021; Arrogante et al., 2016; Aradilla, 2013; Meléndez Chávez et al., 2013; Hernández Rodriguez, 2020). In recent years, emotional intelligence has been gaining great importance in psychology and different organizational fields. ‘It is a variable that is linked to coping styles and the appropriate management of conflictive situations’, which is why it has become a highly relevant topic within organizations to understand how emotions work and how they regulate human behavior in different aspects of life, one of them being the professional sphere (Nespereira-Campuzano and Vázquez-Campo, 2017).

The recent pandemic caused by the new coronavirus created a high demand for health services and, consequently, for the care of healthcare personnel who were confronted for the first time with a situation of this magnitude (De Caneva et al., 2020), which has put many aspects of patient care to the test and, above all, jeopardized the mental health of healthcare professionals at risk (De Caneva et al., 2020; Fletcher and Sarkar, 2013; Ordoñez-Rufat et al., 2021). In addition to the increased workload experienced by these staff in caring for patients infected with this virus, there was a lack of protective measures, reliance on improvised management, and an increase in the perceived risk for these professionals (Puerta Cortés, 2020; Real et al., 2021; Juárez-García et al., 2024; Sánchez Herrero et al., 2022; González Gacel et al., 2021; Celis-Herrero et al., 2021).

During the time between the onset of the pandemic and the different waves experienced by healthcare workers, it is possible that risk perception changed. The understanding of risk perception as the interpretation of a stimulus as threatening, fear of harm, plays a fundamental role as it is not only an uncontrollable fear of contagion that affects the wellbeing and mental health of workers but also affects their decision-making skills, performance, and ability to effectively communicate risk, amongst others. This aggravated the effects of the pandemic in clinical and hospital settings (Juárez-García et al., 2024; González Gacel et al., 2021; Celis-Herrero et al., 2021; Ceberio et al., 2021; Ceberio et al., 2022; Slovic and Weber, 2013; Restrepo, 2016). This variation May be related in important ways to the way in which healthcare staff treat patients affected by the disease. Hence, it is of importance to measure the healthcare professionals’ perception of the pandemic risk (Real et al., 2021; Fletcher and Sarkar, 2013; Ordoñez-Rufat et al., 2021) and the aspects of how healthcare professionals perceived resource management during the COVID-19 pandemic (Aradilla, 2013). In other words, it is important to determine the kind of attitude the professionals had toward the management that was carried out by the authorities and those responsible for acting in the face of the COVID-19 pandemic (Bentler and Bonett, 1980; Cohen, 1988).

Numerous studies, rapid systematic reviews, and meta-analyses were conducted during the outbreak of the pandemic, which focused on assessing certain variables and thus were able to determine the condition of the mental health of frontline health workers, but long-term data could not be obtained (Hernández Rodriguez, 2020; Figueroa Pico et al., 2021; Sánchez Herrero et al., 2022; Shechter et al., 2020; Rodriguez Zambrano, 2020; Maguiña Vargas et al., 2020; Sandin et al., 2021).

The majority of the studies were limited to determining how providing treatment to patients with COVID-19 affected the mental health of healthcare professionals in intensive care units, in-hospital and out-of-hospital emergency departments, etc. However, these studies did not identify whether there are predictor variables such as the professionals’ perception of risk, their attitude toward the management carried out by the administration or even their levels of emotional intelligence during the pandemic and the effect on their psychological wellbeing, and their depressive symptoms. There is also a need to determine the effect of the mediating variables, resilience and discrepancy in the satisfaction of psychological needs, on the mental health of healthcare professionals (Dosil Santamarría et al., 2021; Peñafiel-León et al., 2021; Rodriguez Zambrano, 2020; Lozano-Vargas, 2020; Muñoz Fernández et al., 2020; Arias Molina et al., 2020; Ferrer, 2020; Huarcaya-Victoria, 2020).

According to the broader understanding of mediation, mediating variables explain how a certain event acquires internal psychological meaning and influences certain responses in human beings (Juárez-García et al., 2024; Arenas Sanchez and Pinzon-Amaya, 2021). In this way, we can identify groups that are at a higher risk of experiencing mental health problems and understand the long-term impact of the COVID-19 pandemic on the mental health of certain populations (Hernández Rodriguez, 2020; Xiong et al., 2020; De Juan, 2021; González Gacel et al., 2021).

Therefore, taking into account all the above aspects, the main objective of this study was to test a mediation model, where resilience and psychological needs satisfaction played a mediating role in the risk perception, attitude toward the pandemic management by the administration and emotional intelligence on psychological wellbeing, and depressive symptoms of frontline professionals. In this way, we can identify and highlight mental health problems in healthcare workers, especially emergency workers, and offer strategic information based on evidence-based medicine to provide services to support the psychological wellbeing of healthcare workers in the face of future pandemics (Sánchez Herrero et al., 2022; González Gacel et al., 2021; Celis-Herrero et al., 2021; Casales, 2022; Hernández Rodriguez, 2020; Xiong et al., 2020; De Juan, 2021; Cori et al., 2020; Gerhold, 2020).

## 2 Materials and methods

### 2.1 Study design

A descriptive cross-sectional study was carried out. Healthcare professionals belonging to hospital emergency services, critical care units, and out-of-hospital emergency services in southern Spain were the study participants.

### 2.2 Ethical considerations

This study was conducted in accordance with the tenets of the Declaration of Helsinki. The study was approved by the Ethics Committee of the University of Malaga (masked for peer review). All participants were required to provide their informed consent through explicit digital acceptance before starting the questionnaire. Meeting this requirement was necessary to continue with the completion of the questionnaire. The questionnaire was anonymous, and confidentiality and data protection were guaranteed.

### 2.3 Sampling and procedure

A total of 405 participants (74.3% were female individuals and 25.7% were male individuals) in the age range between 22 and 65 years (*M* = 40, *SD* = 10.57) were included in the study. An online survey was administered to the healthcare professionals dealing with the COVID-19 pandemic. The survey was conducted online and included sociodemographic data such as sex, age, professional category, years of experience in the profession, whether or not they had lived with a person over 60 years of age, and whether or not they had experienced COVID-19, The survey also included the Pandemic Risk Perception Scale (PRPS), the Patient Health Questionnaire (PHQ-9), the Psychological Wellbeing Scale (PWBS), the Trait Meta-Mood Scale (TMMS), the Connor–Davidson Resilience Scale (CD-RISC), the attitude to pandemic management scale (AM), and the basic psychological needs discrepancy scale (BPNDS). The inclusion criteria were as follows: being over 18 years of age and being a healthcare professional working in hospital emergency departments, out-of-hospital emergency departments, intensive care units, and all those units in which they had worked with patients diagnosed with COVID-19 infection. The exclusion criteria were as follows: individuals who did not meet the inclusion criteria mentioned above, those who did not provide informed consent, and those who worked simultaneously in a different unit or in another private hospital. Table 1 provides information about the characteristics of the participants.

**Table 1:** Sample characteristics of the healthcare workers in this study.

Variable	Percentage
Type of healthcare professional	
Doctor	12.6
Nurse	63.2
Nurse’s auxiliary	21.0
Emergency technician	3.2
Living alone	
Yes	14.3
No	85.7
Living with a person older than 60 years	
Yes	26.4
No	85.7
You have been infected with COVID-19	
Yes	19.3
No	80.7

### 2.4 Measures

#### 2.4.1 Sociodemographic questionnaire

A sociodemographic questionnaire is an *ad hoc* tool consisting of questions related to demographic and occupational data with closed questions. The variables included were as follows: sex, age, professional category, years of experience in the profession, whether or not they had lived with a person over 60 years of age, and whether or not they had experienced COVID-19.

#### 2.4.2 COVID-19 pandemic risk perception scale (PRPS)

The questionnaire was developed as an *ad hoc* tool for this research. It assesses the participants’ perception of the risk or danger of the COVID-19 pandemic (Short, 1984; Capone et al., 2021). It consists of 12 items, each with a 5-point Likert-type response (0 = strongly disagree to 4 = strongly agree). The total score ranges from 0 to 48 points; the higher the score, the higher the level of risk perception. In the present sample, the McDonald’s omega (*ω*) value was 0.95.

#### 2.4.3 Patient health questionnaire (PHQ-9)

This is a self-administered diagnostic tool designed to assess the presence of depressive symptoms (Diez-Quevedo et al., 2001; Patel et al., 2019). The questionnaire comprises nine items assessing depressive symptoms experienced over the past fortnight. Respondents rate each item using a 4-point Likert scale, ranging from 0 (indicating no presence of a symptom) to 3 (presence of a symptom almost every day). The total score is calculated by summing all item scores, which can range from 0 to 27. Higher scores indicate greater symptom severity. The thresholds of 5, 10, and 15 indicate low, medium, and high levels of depressive and anxiety symptoms, respectively. In the current sample, the *ω* value was 0.94.

#### 2.4.4 Reduced version of the Spanish adaptation of the psychological wellbeing scale (PWBS)

Based on the Psychological Wellbeing Scale (Díaz et al., 2006; Carol, 1989) and the version proposed by Dierendonck (2004). This scale assesses psychological wellbeing along with six dimensions, including self-acceptance, positive relationships with others, autonomy, environmental mastery, purpose in life, and personal growth. It includes 29 items, which are scored on a 6-point Likert-type (ranging from 1 = strongly disagree to 6 = strongly agree). The maximum score for this scale is 234. Scores above 176 in the total indicate high psychological wellbeing, scores between 141 and 175 indicate high psychological wellbeing, scores between 117 and 140 indicate moderate psychological wellbeing, and scores below 116 indicate low psychological wellbeing. In other words, a higher score indicates higher psychological wellbeing. In addition to the overall score, it is possible to analyze each dimension to consider the predominance of positive effects over negative effects as psychological wellbeing is a multidimensional construct comprising different emotional and cognitive elements. In the present sample, the *ω* value was 0.94.

#### 2.4.5 Reduced version of the trait Meta-mood scale (TMMS)

It is based on the Trait Meta-Mood Scale (TMMS; Salovery et al., 1995). This scale is a short version of the TMMS: the TMMS-12 (Salguero et al., 2009; Salovery et al., 1995). The results of the confirmatory factor analysis corroborate the three-factor structure of the original scale (attention, clarity, and emotional repair); moreover, these dimensions show adequate reliability and correlate with measures of depression, rumination, and vital satisfaction.

The 12-items version of the TMMS is scored on a 6-point Likert-type (1 = strongly disagree to 6 = strongly agree). In the present sample, the *ω* values for attention, clarity, and emotional repair were 0.89, 0.92, and 0.93, respectively.

#### 2.4.6 Two-item version of the Connor–Davidson resilience scale (CD-RISC2)

The two-item version of the Connor*–*Davidson Resilience Scale (CD-RISC2) is based on the Connor*–*Davidson Resilience Scale (2003). The CD-RISC2 consists of items 1 and 8 (scores range from 0 to 8) and was developed as a measure of ‘bounce-back’ and adaptability by the original authors (Vaishnavi and Davidson, 2007). This version includes items 1 (‘Able to adapt to change’) and 8 (‘Tends to bounce back after illness or hardship’). These items attempt to etymologically capture the essence of resilience, i.e., the ability to bounce back and adapt successfully to change. In the present sample, the *ω* value was 0.94.

#### 2.4.7 Attitude to pandemic management scale (AM)

This is an *ad hoc* Likert-type attitude scale. It measures the participants’ attitudes toward the management carried out by the authorities and those responsible for acting in response to the COVID-19 pandemic. It consists of 10 items, each with a 5-point Likert-type response (ranging from 0 = strongly disagree to 4 = strongly agree). The total score ranges from 0 to 40 points, with a higher score reflecting a more favorable attitude toward the management carried out. In the present sample, the *ω* value was 0.88.

#### 2.4.8 Basic psychological needs discrepancy scale (BPNDS)

It is a brief *ad hoc* scale used for the assessment of discrepancy in basic psychological needs. It is based on choice theory (Glasser, 1999; Miranda et al., 2002) and a selection of items used to measure this construct (Miranda et al., 2002). It consists of four items referring to basic psychological needs: belonging, freedom, power recognition, and fun. The participant must evaluate on a 5-point scale (from 0 = I would not like it at all to 4 = I would like to get it to the max) the degree to which he/she would like to achieve what the statement proposes and then score on a similar scale the degree to which he/she currently achieves it (from 0 = I do not get it at all to 4 = I get it totally). In the present sample, the *ω* value was 0.92.

### 2.5 Statistical analysis

First, correlation analysis were carried out between the scores of the predictors, mediators, and dependent variables. Following Cohen’s (Cohen, 1988) criteria, a coefficient of |0.10| was considered a small correlation, |0.30| was considered a moderate correlation, and |0.50| or higher was considered a strong correlation. Subsequently, multiple mediation analyses were carried out. To determine the mediation effects, the procedure described by Hayes (2018) was employed. PROCESS Macros 4.2 in SPSS was used, which involved performing 10,000 bootstrap iterations to generate 90% confidence intervals to test for the indirect effect. One-tailed tests were conducted, given their suitability when directional effects are expected (Cho and Abe, 2013), specifically in mediation analyses (Preacher et al., 2010). The significance of the indirect effect was established when the confidence interval did not include 0 (Rosseel, 2012; Bentler, 1990; Bentler and Bonett, 1980; Browne and Cudeck, 1993; Hu and Bentler, 1999; Hayes, 2018).

**Figure 1:**
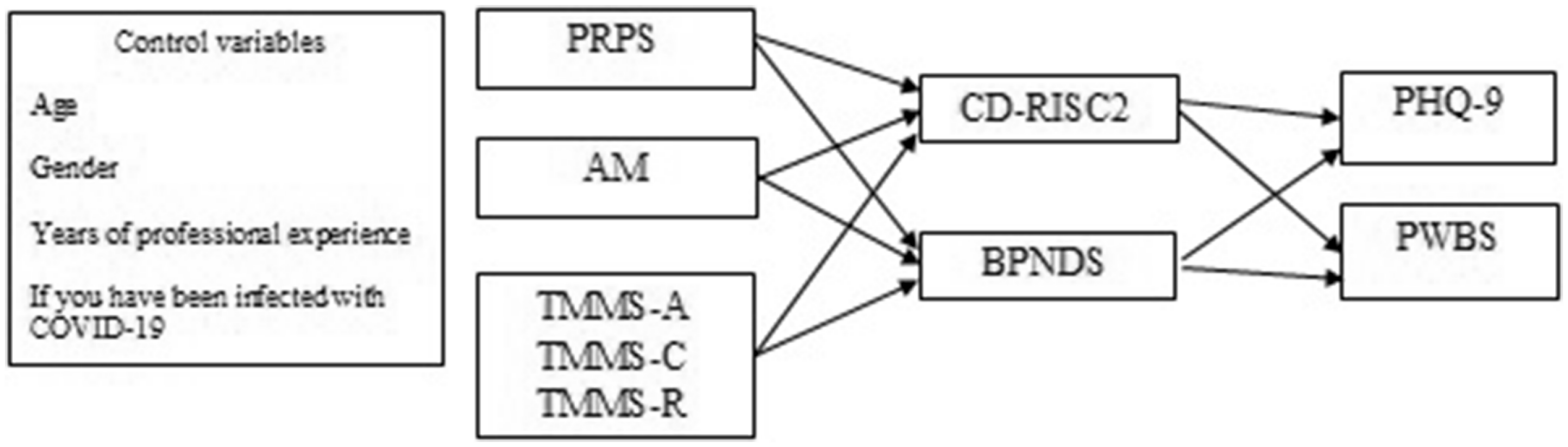
Mediation model tested.

For the mediation analysis, contextual variables were introduced as control variables (age, sex, years of professional experience, and whether you had been infected with COVID-19). Pandemic risk perception (PRPS), attitude to pandemic management (AM), as well as attention, clarity, and emotional repair (TMMS-12) were introduced as predictors, resilience (CD-RISC2) and basic psychological needs discrepancy (BPNDS) as mediators, and depressive symptoms (PHQ-9) and psychological wellbeing (PWBS) as dependent variables (Figure 1). By analyzing the residuals of the model, the assumptions of linearity, normality, and homogeneity of the variances were assessed. All analyses were conducted using SPSS 25 (Diez-Quevedo et al., 2001; Rosseel, 2012; Bentler, 1990; Browne and Cudeck, 1993; Hu and Bentler, 1999).

## 3 Results

After performing Pearson’s correlation analysis for the total scores of the variables considered in the study and the mediation analyses, as mentioned above, the following results were observed:

### 3.1 Correlation analysis

Table 2 shows the Pearson’s correlation coefficient values for the total scores of the variables considered in the study. The results showed that amongst the predictors, only risk perception and attitude to pandemic management had a strong significant negative correlation. On the other hand, the mediator resilience had a weak significant negative correlation with depressive symptoms and a moderate significant positive correlation with psychological wellbeing. The mediator satisfaction of basic psychological needs had a strong positive significant correlation with depressive symptoms and a moderate negative significant correlation with psychological wellbeing.

**Table 2:** Correlation coefficients between the predictors and dependent variables.

	PRPS	AM	TMMS-A	TMMS-C	TMMS-R	CD-RISC2	BPNDS	PHQ-9
AM	−0.60^***^							
TMMS-A	0.01	−0.03						
TMMS-C	−0.03	−0.06	0.40^***^					
TMMS-R	0.01	−0.06	0.33^***^	0.57^***^				
CD-RISC2	−0.09	−0.04	0.13^**^	0.14^**^	0.21^***^			
BPNDS	0.38^***^	−0.29^***^	0.19^***^	−0.06	−0.13^**^	−0.05		
PHQ-9	0.44^***^	−0.36^***^	0.17^***^	−0.05	−0.09	−0.10^*^	0.58^***^	
PWBS	−0.22^***^	0.10^*^	0.18^***^	0.47^***^	0.50^***^	0.35^***^	−0.40^***^	−0.40^***^

*p < 0.05, **p < 0.01, ***p < 0.001.

### 3.2 Mediation analysis

**Figure 2:**
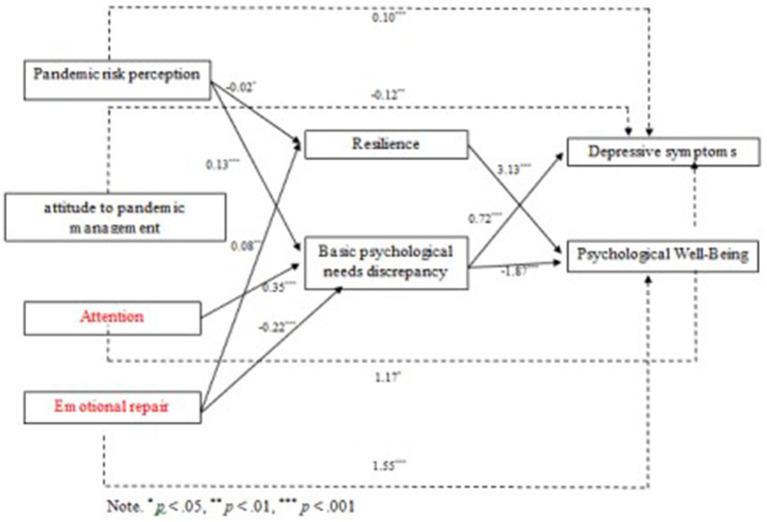
Summary of significant paths in the research model.

The mediation model, which tested to predict the indicators of depressive symptoms and psychological wellbeing, was controlled for four contextual variables (age, sex, years of professional experience, and whether infected with COVID-19) and included perceived pandemic risk, attitude to pandemic management, attention, clarity, and emotional repair as predictors and resilience and basic psychological needs discrepancy as mediators. The regression equations are shown in Table 3, and the significant paths are plotted in Figure 2. With regard to the effects of the contextual variables on the dependent variables, only two variables were statistically significant: younger age was associated with greater psychological wellbeing, and greater professional experience was associated with greater psychological wellbeing.

**Table 3:** Regression equations: contextual variables and depression and psychological wellbeing as dependent variables.

	PHQ-9	PWBS
Contextual variables		
Age	0.02	−0.32^*^
Sex	−0.73	−0.73
Years of professional experience	−0.02	0.42^**^
If you have been infected with COVID-19	−1.34	2.99

^*^p < 0.05, ^**^p < 0.01, ^***^p < 0.001.

In addition, the results showed significant regression coefficients between the following variables: resilience as a dependent variable had a negative relationship with pandemic risk perception (a one unit increase in risk perception decreased resilience by 0.02 units), while it had a positive relationship with emotional repair (for each unit increase in emotional intelligence, resilience increased by 0.08 units); basic psychological needs discrepancy as a dependent variable had a positive relationship with pandemic risk perception (a one unit increase in risk perception produced a 0.13 unit increase in basic psychological needs discrepancy) and attention (a one unit increase in attention produced a 0.35 unit increase in basic psychological needs discrepancy). However, it was negatively related to emotional repair (for each unit increase in emotional intelligence, resilience increased by 0.08 units).

Similarly, depressive symptoms were positively related to basic psychological needs discrepancy (for every one unit increase in basic psychological needs discrepancy, there was a 0.72 unit increase in depressive symptoms). Psychological wellbeing showed a positive correlation with resilience (a one unit increase in resilience resulted in a 3.13 unit increase in psychological wellbeing), while it was negatively related to basic psychological needs discrepancy (for every one unit increase in basic psychological needs discrepancy, there was a 0.22 unit decrease in psychological wellbeing).

In addition, significant and positive direct effects were observed between the predictor pandemic risk perception and depressive symptoms (*β* = 0.10, *p* < 0.001) and attention and depressive symptoms (*β* = 1.17, *p* < 0.05), while a significant and negative direct effect was observed between attitude to pandemic management and depressive symptoms (*β* = −0.12, *p* < 0.01). Emotional repair had a significant and positive direct relationship with psychological wellbeing (*β* = 1.55, *p* < 0.001).

Finally, the mediation analysis showed a large set of significant indirect effects. Pandemic risk perception via basic psychological needs discrepancy had a positive indirect effect on depressive symptoms and a negative indirect effect on psychological wellbeing. In addition, the resilience pathway had a negative indirect effect on psychological wellbeing. Attitude to pandemic management via basic psychological needs discrepancy showed a negative indirect effect on depressive symptoms and a positive indirect effect on psychological wellbeing, while attitude to pandemic management via resilience exhibited an indirect negative effect on psychological wellbeing. Regarding the dimensions of emotional intelligence, attention via basic psychological needs discrepancy showed a positive indirect effect on depressive symptoms and a negative indirect effect on psychological wellbeing. Emotional repair via basic psychological needs discrepancy showed a negative indirect effect on depressive symptoms and a positive indirect effect on psychological wellbeing, while via resilience, it showed a negative indirect effect on depressive symptoms and a positive indirect effect on psychological wellbeing. The results are shown in Table 4.

**Table 4:** Significant spillover effects of the mediation models.

Predictors	Mediators	Dependent variable	Effect	BootSE	Bootstrap 90% CI	
					LL	UL
PRPS	NBPDS	PHQ-9	0.10	0.02	0.06	0.12
		PWBS	−0.24	0.05	−0.31	−0.16
	CD-RISC2	PWBS	−0.07	0.03	−0.13	−0.02
AM	NBPDS	PHQ-9	−0.04	0.02	−0.08	−0.01
		PWB	0.10	0.06	0.01	0.20
	CD-RISC2	PWB	−0.08	0.48	−0.16	−0.01
TMMS-A	NBPDS	PHQ-9	0.29	0.06	0.20	0.39
		PWB	−0.61	0.12	−0.81	−0.42
TMMS-R	NBPDS	PHQ-9	−0.19	0.06	−0.28	−0.10
		PWBS	0.40	0.12	0.19	0.60
	CD-RISC2	PHQ-9	−0.02	0.02	−0.06	−0.01
		PWB	0.26	0.10	0.11	0.45

## 4 Discussion

The COVID-19 pandemic had a major impact and posed a major challenge for healthcare workers. They are often faced with a heavy workload and very demanding conditions. In most cases, they do their work with the feeling that they have few resources and support (Forner-Puntonet et al., 2021; Erquicia et al., 2020; Santamaría et al., 2021). Although this type of pandemic has been shown to cause high levels of different types of symptoms in the workers (depression, anxiety, suicidal ideation, insomnia, etc.), no study has been carried out to identify which variables can mitigate this impact (Erquicia et al., 2020; Santamaría et al., 2021).

The present study aimed to analyze the mediating effect of psychological needs satisfaction and resilience in a sample of healthcare professionals from different hospitals and out-of-hospital emergency services by testing a serial mediation model, controlling for a set of contextual variables of interest in the context of emergency services. For this purpose, linear correlation and mediation analyses were performed.

First, the results of the correlation analysis showed that psychological wellbeing was moderately positively correlated with resilience and moderately negatively correlated with the discrepancy in psychological needs satisfaction. The results obtained were congruent with the empirical evidence found in this field, which concluded that resilience correlates positively and directly with psychological wellbeing (Estrada Araoz and Mamani Uchasara, 2020). Depressive symptoms had a weak negative correlation with resilience and a strong positive correlation with BPN. To further explore these results, a mediation analysis was conducted, which included the following: contextual control variables, pandemic risk perception (PRPS), attitude to pandemic management (AM) and emotional intelligence (TMMS-12) as predictors, resilience (CD-RISC2) and discrepancy in psychological needs satisfaction (Carrillo-García et al., 2016) as mediators, and depressive symptoms (PHQ-9) and psychological wellbeing (PWBS) as dependent variables.

After performing the mediation analysis, significant and positive direct effects were observed between the predictor perception of pandemic risk and depressive symptoms and attention and depressive symptoms, while a direct and significant negative effect was observed between attitude toward pandemic management and depressive symptoms. In addition, emotional repair was found to have a significant and negative direct relationship with psychological wellbeing. These results were consistent with the empirical evidence found in the field (Bermudez et al., 2013; Veliz Burgos et al., 2018).

As for the effects of the contextual variables on the dependent variables, younger age was associated with greater psychological wellbeing and greater professional experience was associated with greater psychological wellbeing. These results were consistent with the results in the literature (García, 2013; Mayordomo et al., 2016; Ballesteros et al., 2006).

The results also showed significant regression coefficients between the variables, such as resilience maintaining a negative relationship with the perception of pandemic risk and a positive relationship with emotional repair. However, the variable, the discrepancy in basic psychological needs satisfaction, had a positive relationship with the perception of pandemic risk and with attention, as well as a negative relationship with emotional repair. In addition, depressive symptoms were positively related to basic psychological needs discrepancy.

Numerous studies have been conducted on the mental health of frontline responders of the pandemic. However, little research has been conducted on the presence of predictor and mediator variables to understand how healthcare workers perceived the pandemic and what consequences it had on their mental health in the short and long term.

No mediation study has been carried out to determine whether there are psychological variables that cushioned the mental health effects experienced during the pandemic by healthcare personnel belonging to critical care units who cared for patients infected with COVID-19 during the different waves of the pandemic (Arrogante, 2014b).

For all these reasons, it is essential to understand which variables are important to know and control in order to be able to mitigate and prevent the effects of periods of stress that May arise during future pandemics and to ensure the adequate mental health of our healthcare personnel.

Once the variables are known and controlled, it will be necessary to incorporate activities into their usual practice, such as mindfulness activities (Brito Pons, 2022; Forner-Puntonet et al., 2021; Erquicia et al., 2020), also known as mindfulness, to reduce the stressors to which healthcare personnel are subjected, in addition to lowering the levels of anxiety, depression, etc. There are also activities such as resilience training and development that would greatly benefit these professionals with low levels of resilience in adapting positively to the characteristics of their work environment. Currently, there are resilience training programs aimed at the general population and specific for healthcare personnel. Such training programs should be promoted in nursing schools and hospitals to improve the clinical practice of these professionals (Arrogante, 2014b; Dunn et al., 2014; Mejillo et al., 2004; Sebri et al., 2024).

It is important to conduct studies on how these therapies affect these personnel, in addition to carrying out studies on the levels of post-traumatic stress and whether suicidal ideation exists and how to prevent it. This approach would ensure adequate mental health of our health personnel in the face of future pandemics (Biggs et al., 2017).

## Data Availability

The original contributions presented in the study are included in the article/supplementary material, further inquiries can be directed to the corresponding author.
